# The relationship of serum gastrin-17 and oral mucositis in head and neck carcinoma patients receiving radiotherapy

**DOI:** 10.1007/s12672-022-00570-6

**Published:** 2022-10-21

**Authors:** Congye Wu, Yehong Liu, Feiyue Shi, Fei Chen, Youcai Zhao, Huanyu Zhao

**Affiliations:** 1 Department of Gastroenterology, Nanjing First Hospital, Nanjing Medical University, Nanjing, China; 2 Department of Oncology and Radiotherapy, Nanjing First Hospital, Nanjing Medical University, Nanjing, China; 3 Department of Pathology, Nanjing First Hospital, Nanjing Medical University, Nanjing, China

**Keywords:** Gastrin-17, Oral mucositis, Radiotherapy, Head and neck carcinoma

## Abstract

**Objective:**

The aim of this study was to analyze the relationship of serum gastrin-17 (G-17) and oral mucositis in head and neck carcinoma (HNC) patients receiving radiotherapy.

**Methods:**

Serum G-17 were detected in patients before and after radiotherapy. Patients were divided into high G-17 group (baseline serum G-17 ≥ 5pmol/L) and low G-17 group (baseline serum G-17 < 5pmol/L). The severity of oral mucositis was analyzed between the two groups. Other complications such as dysphagia, salivary gland, mandible, thyroid function, larynx, pain, and weight loss were also investigated.

**Results:**

Forty-two patients were analyzed in this study. The level of serum G-17 had a significant decrease after radiotherapy (7.29 ± 5.70pmol/L versus 4.93 ± 4.46pmol/L, *P* = 0.038). In low serum G-17 group, the incidences of grade 0, 1–2 and 3–4 of oral mucositis were 0%, 30.4%, and 69.6%, respectively. In high serum G-17 group, the incidences of grade 0, 1–2 and 3–4 of oral mucositis were 0%, 63.2%, and 36.8%, respectively. Pearson correlation analysis showed that serum G-17 was negatively correlated with oral mucositis (r=-0.595, *P* < 0.01). Weight loss of low G-17 group was more serious than that of high G-17 group.

**Conclusion:**

Serum G-17 has a close relationship with oral mucositis. Baseline serum G-17 may be a potential predictor for the severity of oral mucositis in HNC patients receiving radiotherapy.

## Introduction

Annually, nearly 900,000 patients are diagnosed with head and neck carcinoma (HNC) [1]. Although combined methods in modern oncology have made rapid progress, radiotherapy remains a primary treatment for patients with HNC [2]. About 80% of patients need radiotherapy at different stages of treatment. Precise radiotherapy can effectively improve the local control rate and cure rate of the tumor, but the normal tissues such as parotid gland, cerebrospinal cord and cranial nerve around the tumor will receive a certain dose of radiation. Acute complications such as oral mucositis, swallowing disorders, xerostomia and skin pain emerge in varying degrees, leading to impaired quality of life and malnutrition [3]. New technical modalities and biomarkers are critical needed to reduce these complications in HNC [4].

Oral mucositis is a common side effect of chemotherapy and radiotherapy. The incidence is about 85–100% in HNC patients receiving radiotherapy [5]. Previous studies have shown that the severity of oral mucositis is affected by radiotherapy mode, low body mass, prolonged neutrophil recovery, and young age [6, 7]. As oral mucositis can cause reduced food intake, which may affect gastric acid secretory capacity, we initially intended to retrospectively analyze the relationship of gastric functions with food intake. The gastric functions including serum gastrin-17 (G-17), pepsinogen I (PG-1), pepsinogen II (PG-II) and the ration of PG I/PG II have been widely used in clinic. The baseline serum G-17 seemed relatively lower in patients with severe oral mucositis. This study aimed to investigated the relationship of serum G-17 and oral mucositis in HNC patients underwent radiotherapy.

## Methods

### Patients

Between January 2018 to December 2020, 76 HNC patients received radiotherapy at the Departments of oncology, Nanjing First hospital. This retrospective study included patients who detected gastric function before and after radiotherapy. Exclusion criteria included chronic and acute gastric diseases, ECOG score_xx_1, receiving enteral nutrition and/or PPI, and insufficiency of the data. Forty-two patients were left for analysis. Patient demographic data including age, sex, history of smoking, and performance status were recorded on standard admission proformas. Other data, such as tumor characteristics including site and stage distribution, and treatment modalities were supplemented from the hospital laboratory database.

### Treatment modalities

Primary radiotherapy was generally delivered in 5 fractions of 2 Gy each week, for a total dosage of 70 Gy. Chemoradiation was consisted of cisplatin 100 mg/m^2^ every three weeks (days 1, 22 and 43), and concurrent radiotherapy was performed as primary radiotherapy regimen. Postoperative radiotherapy was given at a total dose of 56 to 66 Gy with consideration of pathological risk factors.

### Complications

Acute toxicities were categorized according to the Common Terminology Criteria (CTC) for AEs, version 3.0 (< 90 days posttreatment). The main toxicities included mucosa, skin, dysphagia, pain, weight loss, xerostomia, and larynx.

### Test of gastric function

Serum G-17, PG-I and PG-II were determined by enzyme immunoassay (ELISA) as directed by the manufacturer. Fasting blood samples were taken before and after radiotherapy within 1 week. Fresh samples were dispatched on time to the clinical laboratory and were examined within a few hours. The normal ranges of serum G-17 (1–15pmol/L), PG-I (70–165 ug/L), PG-II (3–15 ug/L), and PG-I/II ratio (7–20) were adopted as reference values. Patients were divided into two groups based on their baseline serum G-17 levels: low group (G-17 < 5pmol /L) and high group (G-17 ≥ 5pmol /L).

### Statistics

Statistical analyses were performed using the SPSS Statistical Software (version 18). Continuous variables in the form of mean ± SEM were analyzed with two tailed *t* test. The proportional compositions of variables were analyzed using χ^2^ test or Fisher’s exact test. Pearson correlation analysis was performed to identify the relationship of serum G-17 and oral mucositis. A *P* value < 0.05 was considered statistically significant.

## Results

### Patients’ characteristics

Among the 42 patients, 33 patients were male. The mean age of the study population was 62.2 ± 13.2 years. The median follow-up for all patients was 15 months. The baseline characteristics of patients before radiotherapy were listed in Table 1. Tumor locations included nasopharynx, oral cavity, oropharynx, hypopharynx and larynx. The mean cumulative radiation dose was 64.8 ± 4.5 Gy, and the mean treatment duration was 45.3 ± 3.8 days.

**Table 1:** The baseline characteristics of patients before radiotherapy

Characteristics	Number (%)
Age	
18–60 years	14 (33.3)
> 60 years	28 (66.7)
Sex	
Male	33 (78.6)
Female	9 (21.4)
Smoking	
Smoker	29 (69.0)
Nonsmoker	13 (31.0)
ECOG performance status	
0	33 (78.6)
1	9 (21.4)
Tumor site	
Nasopharynx	17 (40.5)
Oral cavity	6 (12.3)
Oropharynx	5 (11.9)
Hypopharynx	2 (4.8)
Larynx	12 (28.6)
T stage of primary tumor	
T1	13 (31.0)
T2	15 (35.7)
T3	9 (21.4)
T4	5 (11.9)
Nodal stage	
N0	18 (42.9)
N1	14 (33.3)
N2	6 (14.3)
N3	4 (9.5)
Treatment modalities	
Radiotherapy conventional fractionation	9 (21.4)
Postoperative radiotherapy	12 (28.6)
Chemoradiation	21 (50.0)

### The change of serum G-17

The levels of serum G-17, PG-I, PG-II and the ration of PG-I/PG-II before and after radiotherapy were shown in Table 2. The level of serum G-17 had a significant decrease after radiotherapy (7.29 ± 5.70pmol/L vs. 4.93 ± 4.46pmol/L, *P* = 0.038). No significant changes in PG-I, PG-II and the ration of PG-I/PG-II were observed.

**Table 2:** The serum values of gastric function before and after radiotherapy

	Before radiotherapy	After radiotherapy	*P*
Gastrin-17, pmol/l	7.29 ± 5.70	4.93 ± 4.46	0.038
Pepsinogen I, ug/L	141.44 ± 91.58	123.61 ± 78.82	0.342
Pepsinogen II, ug/L	19.80 ± 13.84	15.14 ± 14.7	0.138
Pepsinogen I/II	10.11 ± 11.83	11.43 ± 11.04	0.533

### The relationship of oral mucositis and serum G-17

The results of oral mucositis and serum G-17 were shown in Table 3. For patients with baseline serum G-17 < 5pmol/L, the incidences of grade 0, 1–2 and 3–4 of oral mucositis were 0%, 30.4%, and 69.6%, respectively. For patients with baseline serum G-17 ≥ 5pmol/L, the incidences of grade 0, 1–2, and 3–4 of oral mucositis were 0%, 63.2%, and 36.8%, respectively. Treatment modalities had no statistical differences between low and high G-17 groups. Low G-17 group had a significantly higher incidence of severe oral mucositis than that of high G-17 group (69.6% vs. 36.8%, *P* = 0.034). For the whole group, the baseline level of G-17 in patients with grade 3–4 oral mucositis was 4.74 ± 3.33pmol/L, which was significantly lower than the result of 9.84 ± 6.47pmol/L in patients with grade 1–2 oral mucositis. Pearson correlation analysis showed that serum G-17 was negatively correlated with oral mucositis (r=-0.595, *P* < 0.01).

**Table 3:** The incidences of oral mucositis between low and high G-17 groups

	Low G-17 group, n = 23	High G-17 group, n = 19	*P*
RF dose, Gy	65.5 ± 8.6	63.5 ± 7.3	> 0.05
Primary radiotherapy	5 (21.7)	4 (21.1)	> 0.05
Postoperative radiotherapy	6 (26.1)	6 (31.6)	> 0.05
Chemoradiation	12 (51.2)	9 (47.4)	> 0.05
Oral mucositis Grade			
1–2	7 (30.4%)	12 (63.2%)	0.034
3–4	16 (69.6%)	7 (36.8%)	

### Other complications

The other short complications after radiotherapy between high G-17 group and low G-17 group were listed in Table 4. There were no significant differences in skin, dysphagia, pain, xerostomia and larynx. The grade 0,1–2 and 3–4 of weight loss in low G-17 group were 3 (13.0%), 12 (52.1%) and 8 (34.8%), respectively. The results in high G-17 group were 5 (26.3%), 11 (57.9%) and 3 (15.8%), respectively. Correspondingly, the magnitude of decrease in body weight loss was higher in low G-17 group than that in high G-17 group (17.9 ± 7.3% vs. 14.7 ± 5.9%, *P* = 0.13).

**Table 4:** The incidences of other complications of radiotherapy between low and high G-17 groups

	Low G-17 group, n = 23			High G-17 group, n = 19		
	Grade			Grade		
	0	1–2	3–4	0	1–2	3–4
Skin	2 (8.7)	13 (56.5)	9 (39.1)	3 (15.8)	11 (57.9)	5 (26.3)
Dysphagia	5 (21.7)	10 (43.5)	8 (34.8)	4 (21.1)	9 (47.4)	6 (31.6)
Pain	11 (47.8)	12 (52.2)	0 (0)	11 (57.9)	8 (42.1)	0 (0)
Weight loss	3 (13.0)	12 (52.1)	8 (34.8)	5 (26.3)	11 (57.9)	3 (15.8)
Xerostomia	6 (26.1)	14 (60.9)	3 (13)	3 (15.8)	13 (68.4)	3 (15.8)
Larynx	18 (78.3)	5 (21.7)	0 (0)	15 (78.9)	4 (21.1)	0 (0)

## Discussion

Oral mucositis is a common complication in almost all HNC patients underwent radiotherapy [2]. It reduces the quality of life and requires more care. How to identify patients who are prone to oral mucositis is crucial in the therapy process. The results of this study show that serum G-17 has close relationship with oral mucositis in HNC patients. Baseline serum G-17 before radiotherapy may be a potential predictor for the severity of oral mucositis.

Serum G-17 is a noninvasive biomarker reflecting the structure and functional status of gastric mucosa [8, 9]. It is only secreted by G cells in the gastric antrum. The secretion is mainly affected by the pH value in the stomach, the number of G cells and food intake. It is a sensitive indicator reflecting the secretion function of gastric antrum and is used for screening and diagnosing atrophic gastritis and gastric cancer [10]. In this study, we observed that the level of serum G-17 decreased after radiotherapy. This may be caused by decreased food intake and atrophy of gastric antrum.

Oral mucositis is an inflammatory reaction characterized by cytokines with a pro-inflammatory profile. Gastrin can promote the growth of normal gastrointestinal mucosa and maintain gastrointestinal mucosal integrity [11]. Barrett’s esophagus is an acquired condition resulting from severe esophageal mucosal injury. According to a prior study, patients with Barrett’s esophagus (BE) had lower serum G-17 levels than non-BE controls [12]. Through the generation of prostaglandin E2 and cyclooxygenase-2 via the CCK-2 receptor, G-17 dramatically stimulates cell proliferation and DNA synthesis [13]. As we know, cyclooxygenases and prostaglandins play key role in mucosal protection in the gastrointestinal tract [14]. Radiation-induced oral mucositis can be less severe if cyclooxygenase-2 expression is suppressed [15]. This may explain why low serum G-17 group has more serious oral mucositis after radiotherapy.

In patients with HNC, weight loss and malnutrition are subsequent diseases [16]. In gastric bypass models, gastrin infusion can prevent mucosal atrophy and attenuate the body weight reduction [17]. In a systematic review, the authors discovered that there was little information available for HNC patients about the effects of nutrition on symptoms and outcomes [18]. However, severe oral mucositis induced by radiotherapy can directly lead to reduced food intake, malnutrition and weight loss. Many studies have investigated in an attempt to reduce mucositis and weight loss [19, 20]. Catrina et al. [19] identified twenty-four studies, honey and the use of benzydamine hydrochloride mouthwash were not advised due to the generally poor quality of the research and the scant evidence supporting the intervention. Although the authors suggested glutamine as a method to lessen oral mucositis, there is still a need for high-quality studies with a widely accepted technique to help reduce heterogeneity. In a multicentric randomized study, Sanctis et al. [21] observed that radiation-induced oral mucositis was not relieved by *Lactobacillus brevis* CD2. More studies about salivary microbiota are required. According to Amanda et al. [22], photobiomodulation for oral mucositis has beneficial effects on preventing weight loss and reducing body mass index in HNC patients who received chemoradiotherapy. Generally, relieving oral mucositis can improve weight loss and nutritional condition.

This study has some limitation that should be noted. The limited sample size needs more work to be done to verify and confirm the results. however, this small sample size is also meaningful because it harkens back to a more inspiring trend. The result can inspire further researches on how to alleviate oral mucositis in HNC patients receiving radiotherapy. Gastroscope combined with serum G-17 before radiotherapy may be used to assess patients’ gastric function and risk of oral mucositis.

In conclusion, serum G-17 has a close relationship with the severity of oral mucositis. Identifying patients who are prone to oral mucositis before radiotherapy is helpful to devote more attention to care and make nutrition plan for NHC patients. Baseline serum G-17 may be a potential predictor for the severity of oral mucositis in HNC patients receiving radiotherapy.

## Data Availability

The datasets used or analyzed during the current study are available from the corresponding author on reasonable request.
