# Correction: The relationship of serum gastrin-17 and oral mucositis in head and neck carcinoma patients receiving radiotherapy

**DOI:** 10.1007/s12672-023-00621-6

**Published:** 2023-02-02

**Authors:** Congye Wu, Yehong Liu, Feiyue Shi, Fei Chen, Youcai Zhao, Huanyu Zhao

**Affiliations:** 1 Department of Gastroenterology, Nanjing First Hospital, Nanjing Medical University, Nanjing, China; 2 Department of Oncology and Radiotherapy, Nanjing First Hospital, Nanjing Medical University, Nanjing, China; 3 Department of Pathology, Nanjing First Hospital, Nanjing Medical University, Nanjing, China

**Correction** **:** **Discover Oncology 13: 110 (2022)** **https://doi.org/10.1007/s12672-022-00570-6**

The original publication of this article contained an incorrect author name. The incorrect and correct information is available in this correction article. The original article [1] has been updated.

- The incorrect author name is: Yongcai Zhao
- The correct author name is: Youcai Zhao
